# How do women respond to increased care needs of their parents? The economic costs of informal caregiving^☆^

**DOI:** 10.1016/j.jeoa.2025.100589

**Published:** 2025-10-25

**Authors:** Jacek Barszczewski, Ricarda Milstein, Jinkook Lee, Ana Llena-Nozal

**Affiliations:** a OECD, France; b University of Southern California, USA

**Keywords:** Informal caregiving, Labour supply, Long-term care, J14, J22, I10

## Abstract

When a person becomes care-dependent, family members often provide informal care. This paper examines the economic impact of informal caregiving, with a particular focus on women who care for their ageing parents. Using data from the Survey of Health, Ageing and Retirement in Europe (SHARE), covering 24 countries, we estimate the effect of caregiving on labour supply and quantify its associated costs in terms of Gross Domestic Product (GDP). Our findings confirm that women typically respond to worsening parental health by increasing their provision of informal care. Women with parents in poor health have a 5% to 13% higher probability of providing informal care, depending on the region of Europe. Furthermore, our results show that women who take on caregiving responsibilities experience significant reductions in labour supply, especially in Southern, Western, and Central/Eastern European countries, with reductions ranging from 30% to 70%. We also show that the economic cost of these reductions in labour supply is considerable, with a GDP reduction ranging from 0.37% in Western Europe to approximately 0.45% in Southern and Central/Eastern Europe. These results highlight the significant economic consequences of informal caregiving and stress the need for policy measures that support reconciling caregiving with labour market participation. Expanding formal long-term care systems, providing caregiver support, and investing in healthy ageing policies could help address these economic pressures.

## Introduction

Across the Organisation for the Economic Co-operation and Development (OECD), populations are ageing (OECD, 2023), resulting in an increasing need for care to support older people in their (instrumental) activities of daily living, such as bathing, getting dressed, and shopping. This support can be provided by professional formal carers or informal carers, such as family members, friends, and neighbours. Informal carers play a key role in providing care to people in need. Around 60 % of people across the OECD that required some form of care rely exclusively on informal carers (OECD, 2023). Governments often view informal care as an attractive alternative to expanding formal long-term care systems. However, unlike formal care cost, which can be assessed using administrative data, the economic cost of informal care remains difficult to quantify.

From an economic point of view, informal care arrangements might seem attractive for countries that search for policy solutions to cope with increasing need for care amidst a shrinking formal care workforce and fiscal constraints. By reducing reliance on formal caregiving structures, informal care can lower the direct costs of long-term care from the state budget perspective. On the other hand, reliance on informal care also carries a significant cost. Informal carers, particularly those active in the labour market, often struggle to balance their paid work and informal caregiving responsibilities. As a result, some of them respond by reducing their working hours or fully resigning from paid work. This leads to indirect economic costs, such as lower or lost labour income, and limited skill and career development, affecting their wages and consumption. These effects, in turn, lead to lower tax and welfare contributions (Kolodziej et al., 2018). For people with low socio-economic status, reduced pension contributions can increase the risk of poverty in the future, which adds pressure to the welfare system. Moreover, reliance on informal care undermines policy efforts to boost female labour market participation as a response to the shrinking labour force (Carrino, Nafilyan, & Avendano, 2022; He & McHenry, 2015). Quantifying both costs can help policymakers to better reflect on the trade-offs between formal and informal caregiving.

In this paper, we address this issue, by quantifying the economic impact of caregiving among daughters who are the most common type of informal carers. We follow a three-step approach: Firstly, we estimate the daughters’ reduction in labour supply in response to worsening parental health and taking-up caregiving duties. Secondly, we investigate how the effect varies between countries with different formal long-term care structures. Finally, we approximate the cost of labour supply reductions on the economy, measured in terms of Gross Domestic Product (GDP).

This paper builds on well-established research that has documented a negative effect of caregiving on labour market supply (Crespo & Mira, 2014; Kolodziej et al., 2018; Maestas et al., 2024). The negative impact ranges from a reduction in the number of hours worked (intensive margin) (Van Houtven, Coe, & Skira, 2013; Schmitz & Westphal, 2017) to completely withdrawing from the labour market (extensive margin) (Kolodziej, Reichert, & Schmitz, 2018; Nguyen & Connelly, 2014; Van Houtven, Coe, & Skira, 2013).

The significance and magnitude of the effect depends on the intensity of caregiving, the caregiver’s gender, and the availability of formal care. Previous studies have identified a significant impact of *daily* caregiving, or caregiving exceeding *20 h per week* (Carmichael & Charles, 1998; Ciccarelli & Van Soest, 2018; Heitmueller, 2007). The effect is generally weaker or insignificant for caregiving at lower intensity (Carmichael & Charles, 1998; Ciccarelli & Van Soest, 2018; Heitmueller, 2007). In addition, the effect is more pronounced among women, who are more likely than men to take on daily caregiving responsibilities, whereas men tend to provide care less frequently. Since the impact of caregiving on labour supply is stronger among daily carers than those providing care less intensively, this gender disparity further amplifies the effect (Nguyen & Connelly, 2014). As a result, women tend to reduce their labour market participation more strongly (Ciccarelli & Van Soest, 2018; Maestas et al., 2024).

The labour market consequences of informal caregiving also may vary by country. The labour supply responses are likely to be different depending on availability of formal care and support towards informal carers in the form of, e.g., leave and cash benefits, but also on the labour market opportunities. Previous research has identified higher levels of informal care in countries with less availability of formal care (Crespo & Mira, 2014; Kolodziej, Reichert, & Schmitz, 2018). This pattern, referred to as the *North-South gradient*, indicates that Nordic countries have greater availability of formal care and thus lower levels of informal care than their Southern counterparts. Paired with the impact of informal caregiving on labour market supply and welfare contributions, this dynamic can further weaken Southern economies, which already have lower GDP levels.

In this paper, we study *the economic impact of women becoming caregivers,* extending existing literature in a twofold way. First, we extend the time horizon by broadening the analysis over an extended period and increasing the number of countries, further exploiting the heterogeneity in labour markets and the availability of formal care across countries. Previous studies often relied on cross-sectional data or a limited number of survey waves from a small set of countries (Crespo & Mira, 2014; Heger & Korfhage, 2018). We enrich this literature by using data from six waves of the Survey of Health, Ageing and Retirement in Europe (SHARE), covering 24 countries. The broader dataset allows for more robust analyses due to a larger sample size, greater regional variation with the inclusion of Central and Eastern European countries, and a panel structure that tracks individuals over time. Second, we quantify the economic effect of becoming a caregiver. There are few studies on the ‘price tag’ of informal caregiving, and existing research is often inconclusive. While some studies have identified a negative impact of informal caregiving on wages and productivity (Carmichael & Charles, 2003; Skira, 2015; Van Houtven et al., 2013), fewer studies consider the total costs, including reductions in welfare contributions, leading to an underestimation of economic costs.

Based on this literature, three hypotheses guide our analysis. *First*, we expect our analyses to confirm existing research that has found reductions in labour supply among women who become caregivers while still participating in the workforce. *Second*, we anticipate a stronger reduction in labour supply in countries with less availability of formal care structures compared to others. *Third*, we expect a significant impact of reductions in labour supply on the economy as expressed through a reduction in GDP, since we expect a considerable part of the working-age population to decrease their labour participation when becoming carers that provide care on an intensive basis.

The rest of the paper is organised in four sections. The next section presents our data and methodology. Following section details the results of our analysis, followed by a section including discussion of our findings, policy considerations, and limitations. The last section concludes with a summary and suggestions for further research.

## Data and methods

### Estimation sample

In this paper, we use the SHARE data (SHARE-ERIC, 2024b,c,d,e,f,g). The survey was launched in 2002, and data were subsequently collected in 2006, 2008, 2010, 2012, 2014, 2016, and 2019. Due to data limitations, we use wave 1–2 and 4–6 and 8^1^ in this study. This combined dataset includes information on caregivers in 28 European countries and Israel, covering personal characteristics such as age, education, and income, caregiving duties, and employment status, as well as information on their parents’ health. A detailed description of the dataset and comprehensive documentation is available on the website of SHARE (SHARE-ERIC, 2024a).

We limit our sample to 24 countries due to sample size constraints. Following Crespo and Mira (2014), we group these countries in our sample into four distinct geographic clusters, them being: Northern European OECD countries (hereafter ‘the North’) – Denmark, Finland, the Netherlands, and Sweden; Western European OECD countries (hereafter ‘the West’) – Austria, Belgium, France, Germany, Luxembourg, and Switzerland; Southern European OECD countries (hereafter ‘the South’) – Greece, Italy, and Spain; and Central and Eastern European OECD countries (thereafter ‘the Centre/East’) – Bulgaria,^2^ Croatia,2 Czechia, Estonia, Hungary, Latvia, Lithuania, Poland, Romania,2 Slovakia and Slovenia. We expand Crespo and Mira’s (2014) classification by adding the Centre/East as a fourth country group.

We apply several additional restrictions to our sample. Across all four geographic clusters, women are more likely than men to provide intensive forms of care (see Table 8 in Annex B) The difference is the biggest in the South, where women are nearly three times more likely to provide intensive care to their parents than men. In contrast, the smallest difference is observed in the Centre/East, where women are only 27 % more likely to engage in intensive caregiving. Given this, we restrict the sample to women, as intensive caregiving participation was found to lead to a stronger response in labour market supply.

Further, we include only women aged 50–60 to avoid confounding results from simultaneous childcare responsibilities and to ensure that participants have not yet reached the official retirement age, which is set at 60 in several countries (OECD, 2023). We also restrict our sample to women with at least one living parent in good health at the first appearance in the sample. We do so to properly model the effect of a parental health deterioration from good health^3^ that does not require intensive caregiving, to poor health, requiring formal or informal caregiving. We do not restrict our sample to daughters living in the same household to also include informal carers that live within a certain distance to mirror different living arrangements. These restrictions follow Crespo and Mira (2014), allowing for a direct comparison of results.

### Estimation strategy

The impact of caregiving on labour market participation is estimated using the approach proposed by Crespo and Mira (2014). The causal identification is based on estimating treatment effects using a binary instrument. In our study, the treatment effect is daily or weekly informal caregiving^4^ ($IC_{it}$) and the instrumental variable (IV) is poor parental health ($PH_{it}$). $IC_{it}\left(PH_{it}\right)$ represents the potential caregiving decision of a woman i at time t based on the health status of her parents. $LS_{it}\left(IC_{it}=1\right)$ and $LS_{it}\left(IC_{it}=0\right)$ define the potential labour supply decision of a woman i at time t if she were to provide care, or not. Both outcomes of caregiving and labour supply decisions are called potential as they are not observed together for the same individual and at the same point in time.

Every woman in the population belongs to one of four compliance types (Angrist, Imbens, & Rubin, 1996; Crespo & Mira, 2014): *always takers* $\left(IC_{it}(1)=IC_{it}(0)=1\right)$ provide care independent of their parents’ health status, *never takers* ($IC_{it}(1)=IC_{it}(0)=0$) do not provide care independent of their parents’ health status, *compliers* ($IC_{it}\left(1\right)=1,IC_{it}\left(0\right)=0$) provide care only if one of their parents is in bad health, and defiers $\left(IC_{it}(1)=0,IC_{it}(0)=1\right)$ provide care only if both parents are in good health. Parental health $\left(PH_{it}\right)$, is a valid instrument if it is monotone (so there are no defiers), exogenous (parental health status is not correlated with unobserved determinants of employment and caregiving decisions of daughters), and meets the exclusion restriction condition (labour supply decision is independent of parental health once caregiving is accounted for). Monotonicity is a plausible assumption, as it is reasonable to expect that parents’ utility from their daughters’ caregiving increases as their health deteriorates. The exogeneity assumption, however, may be violated if parents’ health capital is correlated with their daughters’ human capital, which is a likely scenario. To address this, control variables in the caregiving and labour supply equations should be carefully selected to approximate daughters’ human capital. Lastly, the exclusion restriction holds if formal care purchased by daughters is negligible, if financial or non-financial transfers from parents to daughters in exchange for caregiving are insignificant, and if parental health has a negligible effect on the geographic distance between parents and daughters. The empirical plausibility of these three assumptions is further discussed in section IV of Crespo and Mira (2014).

In our setting, the treatment regressor is binary (whether a woman provides care or not), and the instrument is also binary, and we assume that it has a monotone impact on treatment. Consequently, an IV estimate can be interpreted as a local average treatment effect (LATE) specific to the instrument. More formally, the LATE parameter for woman i at time t with characteristics $X_{it}$ is given by:

$$\begin{array}{l} \beta \left(X_{it}\right)=E\left[LS_{it}(1)-LS_{it}(0)|IC_{it}(1)-IC_{it}(0)=1,X_{it}\right]\\ \;=\frac{\int \;\left[E\left(LS_{it}|PH_{it}=1,X_{it}\right)-E\left(LS_{it}|PH_{it}=0,X_{it}\right)dF(x)\right.}{\int \;\left[E\left(IC_{it}|PH_{it}=1,X_{it}\right)-E\left(IC_{it}|PH_{it}=0,X_{it}\right)dF(x)\right.}, \end{array}$$

which is the average effect of caregiving on work within the subpopulation of compliers (women whose caregiving decision depends on the parental health instrument). In this context, both the numerator and denominator are of interest. The numerator represents the marginal effect of poor parental health on the labour supply decision of potential caregivers. Similarly, the denominator represents the marginal effect of poor parental health on the caregiving decision, which can be interpreted as the share of compliers in the population.

Both marginal effects (numerator and denominator) can be obtained using parametric approximation from the seemingly unrelated probit regression, using parental health as a regressor. The probit function for labour supply and caregiving is as follows:

$$Pr\left(y_{it}=1\right)=\Phi \left(\beta_{0i}+\beta_{1t}+\beta_{2}PH_{it}+\beta_{3}X_{it}\right),$$

where $y_{it}$ is a binary outcome for working/caregiving for an individual i at time t, and Φ() is the normal cumulative distribution function. $PH_{it}$ takes the value of 1 if at least one parent of individual i at time t is in poor health. $X_{it}$ is a vector of control variables. Estimates from this regression are used to calculate the marginal effect of poor parental health on labour supply and caregiving decisions.

Due to better data availability, we enrich the framework proposed by Crespo and Mira (2014) by estimating the parametric approximation of the numerator and denominator using panel data and individual and time fixed effects. This approach allows us to control for all time-invariant observable and unobservable characteristic that might impact caregiving and labour supply decisions, such as labour market attachment, education, and number of children. Additionally, we control for time fixed effects, such as the phase in the economic cycle. For the panel data estimates, we employ a linear model as there is no established methodology to obtain marginal effects with fixed-effects logit and probit models.

Finally, we perform a back-of-the-envelope calculation to approximate the financial costs of informal caregiving on the economy. We do this in several steps. First, we use survey data to estimate the share of women aged 50–60 affected by parental health deterioration (at least one living parent in poor health). Next, we use these estimates to calculate the number of women who leave the labour market due to caregiving duties and relate this number to total employment. This gives us the change in total employment due to caregiving. Finally, using the elasticity between GDP and employment provided by the European Central Bank (2016), we calculate the equivalent of this change in the GDP terms.^5^ In our back-of-the-envelope calculations, we adopt a market perspective, treating informal care as having a negative impact on GDP. However, this does not imply that providing care informally is inherently unproductive. The estimated value is calculated as a cost from a market standpoint, but from a household perspective, it could instead be viewed as added value.

We conduct four robustness analyses to test the validity of our results. First, we test a different clustering of countries. In this paper, we have expanded on the clustering based on geographic criteria by Crespo and Mira (2014). However, a classification based on geographic criteria might not capture differences in access to formal long-term care and could introduce within-group variation that confounds our results. Therefore, we propose an additional clustering of countries based on key characteristics of formal long-term care systems, which might influence potential caregiving decision and thus impact the labour supply of informal carers. Existing classifications by Ariaans, Linden, and Wendt (2021) or Kraus et al. (2010) were found to be too limited in the number of indicators and cover only some dimensions of long-term care systems, which has prompted the OECD to develop its own methodology (OECD, 2024). This new methodology includes a wide set of indicators (19 measures) in order to cover additional dimensions of long-term care systems, them being governance, financing, quality, availability and access. The OECD methodology synthesises results of four different clustering algorithms to ensure robustness of the final classification. Details on the selection of the indicators and the methodological approach are available in Annex A.

This results in three clusters. Cluster 1 consists of countries with, on average, the most developed long-term care systems. This cluster comprises Austria, Belgium, Denmark, Finland, Germany, Luxembourg, Netherlands and Sweden. Cluster 2 groups Czechia, Estonia, France, Hungary, Lithuania, Italy, Slovakia, and Spain. Long-term care systems in Cluster 2 are, on average, well-developed, but usually experience difficulties in some of the dimensions. For example, Estonia lags behind in funding, while France scores low in access to long-term care. Countries that belong to the last cluster, Cluster 3, are Bulgaria, Croatia, Latvia, Greece, Poland, Romania, and Slovenia. These countries have less-developed long-term care systems, often facing challenges across multiple dimensions. The long-term care system in Greece performs below average across all dimensions except funding, while long-term care in Bulgaria is characterised by low scores in all dimensions except governance.

The classification of countries based on geographic criteria closely aligns with the OECD typology, showing minimal differences. Cluster 1 predominantly includes countries from the North and West regions. Clusters 2 and 3 are primarily composed of countries from the South and Centre/East regions. However, countries in Cluster 2 generally have more developed long-term care systems compared to those from the same regions classified in Cluster 3. A detailed comparison of these two typologies is provided in Annex A.

## Results

### Descriptive statistics

Our sample consists of 7,333 women-year observations, or 5,576 unique women observations. Table 1 presents descriptive statistics of key variables in our sample. The first insight is that a minority of women in our sample are *daily* caregivers. The rate of daily caregiver is highest in the South, with 13 % providing care on a daily basis. This is approximately four times higher than in the North, the country group with the lowest rate of women providing care on a daily basis (3 %). The share of *weekly* caregivers is more similar across the four regions and ranges between 18 % and 22 %.

The share of women participating in the labour force while providing informal care is highest in the North, with female labour supply at 88 %, and lowest in the South with 54 %. The share of women carers with upper secondary or higher education is similar in the North, West, and Centre/East (81 %–86 %), but significantly lower in the South (50 %). The median nonwage income is lowest in the Centre/East despite this country group having the highest share of women with upper secondary or higher education. The nonwage income amounts to less than one fifth of the nonwage income in the North, which records the highest median nonwage income in our sample. Women in the South are most likely to be co-residents of the older person and to live less than 5 km away, followed by women in the Centre/East. Women in the Centre/East have slightly fewer siblings compared to those in other regions. Both women and their parents in this region tend to be in worse health than those in other country groups.

Table 2 shows women’s formal labour market supply depending on their parents’ health status and whether they provide caregiving on a daily basis. It shows considerably lower labour market participation in the South compared to the North, West and Centre/East. Even if both parents are healthy, women in the South are less likely to work than caregivers in any other country group who provide care to at least one parent in poor health on a daily basis. Labour supply is generally lower among people with at least one parent in poor health, and even lower among those who provide daily care, except in the South. The largest difference in labour supply depending on parents’ health is seen in the North, while the smallest is in the South.

The differences in labour market supply presented in Table 2 may partially be explained by differences in observable characteristics between those providing and not providing care. On average, caregivers tend to be slightly older, more likely to be married, and have a lower likelihood of attaining upper secondary or higher education. Importantly, caregivers are, on average, twice as likely to report poor health compared to those with both parents in good health. However, they are 25 % less likely to report poor health than individuals with at least one parent in poor health who do not provide care. This suggests a potential correlation between the health status of caregivers and care recipients. Additionally, caregivers tend to have fewer siblings on average, indicating that being an only child significantly increases the likelihood of providing care. Proximity to parents also plays a crucial role – caregivers are nearly four times more likely to live within 5 km of their parents compared to non-caregivers. Detailed demographic characteristics by parental health and caregiving intensity are provided in Table 9 in Annex B.

### Estimation results

Table 3 presents the estimation results for the employment effect, care effect, and the LATE parameter, replicating Crespo and Mira (2014) with a pooled sample. The estimation is conducted with time fixed effects only (Model 1), with control variables (Model 2), and with control variables and a lag of labour supply (Model 3). Control variables are age dummies, a higher education dummy, and the number of sisters, which approximates the availability of other potential informal caregivers.

Estimates for Model 3 show that the health deterioration of at least one parent has a significant negative impact on women’s employment in all regions except the North. In the North, although the effect is still negative, it is statistically insignificant, likely due to the smaller sample of caregivers in that region. The regression results also indicate that worsening parental health increases the likelihood of women taking on caregiving responsibilities across all four regions. The estimates represent the proportion of compliers – women who provide care once their parent is in poor health. The share of compliers is the highest in the South and the lowest in the North. The last column presents the estimates of LATE parameters, indicating the effect of caregiving on labour supply among compliers. The effect is significant in three regions: West, South, and Central/East, with the probability of reducing labour supply due to the caregiving duties ranging from 30 % to 70 %.

Table 4 displays the results of the replication of Crespo and Mira (2014) using a pooled sample with time fixed effects only (Model 1), with control variables (Model 2), and with control variables a lag of labour supply (Model 3) using the OECD typology of three clusters. The findings indicate a negative and significant effect of parental health deterioration on labour supply in Clusters 2 and 3. However, the effect for Cluster 3 is insignificant, which may be largely attributed to the significantly smaller sample size in this cluster as mentioned above. Regarding caregiving, the results show a positive and significant effect across all three clusters. This effect is stronger in Clusters 2 and 3, which represent countries with less developed long-term care systems compared to those in Cluster 1.

The LATE parameter estimates reveal a significant negative impact of informal caregiving on labour market participation among daughters whose parents experience a decline in health. Interestingly, the LATE parameter is highest in Cluster 1 and lowest (and insignificant) in cluster 3. This pattern suggests that while fewer individuals in countries with well-developed long-term care systems choose to provide care for their parents when needed, those who do are more likely to exit the labour market. This trend may be driven by the fact that these countries not only have more developed formal long-term care systems but also offer more robust support policies for informal caregivers.

Table 5 presents the results of the panel estimation using the geographic grouping based on Crespo and Mira (2014). The overall directions are consistent, with results largely insignificant in the panel estimation compared to the pooled estimates in Table 3 and Table 4. The effect of parental health deterioration on labour supply is now only weakly significant for the South, and shows a negative effect, whereas it remains insignificant for all other geographic areas. The effect of the health deterioration on caregiving is significant for both the South and the West, with a positive direction in both instances. The effect among compliers is only significant in the South, where women who decide to become caregivers are 44 % less likely to participate in the labour market. The panel regression based on the OECD grouping yields similar results to the one based on Crespo and Mira’s (2014) grouping, with only the effect of health deterioration on caregiving being more weakly significant in Cluster 2. Detailed results are presented in Table 11 of Annex B.

These results suggest that there might be unobserved time-invariant factors influencing both caregiving and labour supply decisions that confound the results in Table 3 and Table 4. However, the estimates of the linear probability models with fixed effects should be carefully interpreted, as one concern is that linear probability models can suffer from bias and inconsistency issues (Horrace & Oaxaca, 2006). Nevertheless, a comparison between the pooled probit results in Table 3 and the linear probability model results in Table 10 of Annex B shows no significant differences, suggesting that the choice between linear and nonlinear models does not drive the observed results.

There are alternative explanations for the observed differences between results obtained from pooled probit and fixed-effect model. First, the average number of observations per woman is only 1.319, which is relatively low. This limits the effective sample size for the panel fixed-effects estimation, reducing the precision of the estimates compared to the pooled probit model. Second, the between-individual standard deviation (0.342) is substantially larger than the within-individual standard deviation (0.155), indicating that much of the variation in parental health, which is critical for identifying effects in pooled estimations, is lost when individual fixed effects are applied. These findings support the use of pooled estimates to capture the cost of informal caregiving, as they better leverage the between-individual variation and provide more precise estimates.

Table 6 reports the results of the estimated effect of informal caregiving on total employment in GDP terms. The share of women aged 50–60 with at least one parent in bad health is similar across all four geographic regions, ranging from 12 % in the Centre/East to 15 % in the West. Using the estimates from Table 3, we obtain the change in employment associated with informal caregiving. The employment effect is smallest in the North, with a statistically insignificant reduction of −0.13 %, compared to the South and Centre/East, where it is largest at −0.28 %, and roughly similar to the West. This is equivalent to a 0.21 % reduction of the GDP in the North, whereas it is around twice as high in the other regions. There are only minor differences between the West, South, and Centre/East.

## Discussion

Our results indicate a reduction in labour supply among women when they become caregivers, with responses largely insignificant in the North (likely due to the small sample) but significant in the other regions. This suggest that there are considerable costs to societies attached to informal caregiving.

Our findings show that a share of women withdraw from the labour market when taking on caregiving roles. This confirms our first hypothesis, in which we expected our analyses to confirm existing research that has found reductions in labour supply among women who become caregivers while still participating in the workforce and echoes the findings of results by, e.g., Crespo and Mira (2014), Ciccarelli and Van Soest (2018), Kolodziej, Reichert and Schmitz (2018), and Bolin, Lindgren and Lundborg (2008). These effects are not exclusive to SHARE data, which was used by these authors, but are also consistent with findings using other data sources such as EU-SILC (Mozhaeva, 2021), the Household Income and Labour Dynamics in Australia survey (Nguyen & Connelly, 2014), and responses from the US Survey of Income and Program Participation linked with Social Security Administration (Maestas et al., 2024). The results of our pooled logit regression show significant labour supply reduction in the West, South, and Centre/East regions following parental health deterioration, while results in the North are insignificant. This may be due to the availability of generous formal care in the North. With these results, we cannot reject our second hypothesis, in which we anticipated a stronger reduction in labour supply in countries with less availability of formal care structures compared to others. This is in line with the results of Kolodziej, Reichert and Schmitz (2018), who found reduced labour supply impacts in countries with more robust formal long-term care structures. Similar patterns are observed in individual countries and subsets of countries. Mozhaeva (2021) finds a negative effect of informal caregiving on labour supply in the three Baltic states Estonia, Lithuania, and Latvia, which could partly be explained by a lower availability of formal long-term care. In contrast, Gautun and Bratt (2024) do not find an effect of caregiving on labour supply in Norway, which they linked to the large availability of formal care and generous sick leave policies in this country. Perdix and Roquebert (2021) find that an increase in formal care is associated with a small decrease in informal care in France, which could in turn lead to a smaller effect on reductions in labour supply.

Results of our panel estimates show a largely insignificant effect of changes in parental health on labour supply and caregiving, which may suggest a selection effect in our sample. Daughters who are less attached to the labour market are more likely to provide care for their parents. The impact of labour market attachment is neutralized by fixed effects, However, the results from fixed-effects estimators should be interpreted with caution, as the sample size and variation used to identify the effect in the panel data estimation are significantly smaller than in the case of pooled regressions.

The clustering of countries based on characteristics of long-term care systems, as developed by the OECD (Llena-Nozal et al., 2025), yielded results that differ slightly from those based on geographic grouping proposed by Crespo and Mira (2014). This indicates that decisions about caregiving and labour supply are affected by factors like characteristics of long-term care systems. In countries with more developed long-term care systems, daughters are less likely to take on caregiving responsibilities for their parents. However, when they do assume these roles, they are more likely to withdraw from the labour market. This pattern may be attributed to the availability of better benefits and support systems for informal caregivers in such countries. That being said, these findings do not exclude the potential influence of other factors, such as social norms, legal obligations, and labour market opportunities, on caregiving decisions. Previous studies have highlighted the importance of these elements in shaping caregiving behaviour (Bakx, de Meijer, Schut, & van Doorslaer, 2014; Mozhaeva, 2021; Maestas, Messel, & Truskinovsky, 2024).

Our back-of-the-envelope analysis indicates a pronounced economic penalty for societies resulting from women reducing their labour supply when becoming informal carers. These results confirm our third hypothesis, in which we assumed a significant impact of reductions in labour supply on the economy as expressed through a reduction in GDP, since we expected a considerable part of the working-age population to decrease their labour participation when becoming carers that provide care on an intensive basis.

The penalty from women reducing their labour supply in GDP terms is lowest in the North (insignificantly different from zero), likely due to the large availability of formal care structures and generous sick leave policies help to mitigate effects from the deterioration of parental health on potential caregivers. The limited effect on labour supply and GDP aligns with our descriptive statistics in Table 1, which showed much lower rates of daily caregiving in the North, but less of a difference in weekly caregiving between the North and other regions.

Our findings suggest significant economic costs when compared to the average spending on long-term care across the OECD, which stands at 1.8 % of the GDP (OECD, 2023). This indicates that the cost of reductions in labour supply due to caregiving in GDP terms almost matches these countries’ spending on formal long-term care. Economic costs from informal long-term care limit countries’ ability to invest in health and long-term care. The OECD calculations suggest that countries would need to invest an additional 1.4 % of their GDP annually to make their health systems resilient to crises, such as a global pandemic (OECD, 2023). Losses from reductions in labour supply due to becoming a caregiver equate to around one third of these investments.

Other institutions, such as European Comission (2021) find a somewhat larger effect of 1.05 % of GDP. Our results differ due to different assumptions and our more conservative approach. For example, European Comission focuses only on the state budget, whereas we look at the total economic cost. We account not only for the direct cost of carers leaving the workforce but also for the indirect costs resulting from spillover effects associated with their exit from employment, for example a negative effect on consumption.

Moreover, our results are smaller than those obtained from calculating total costs of informal care. For example, Ekman et al. (2021) estimated the total costs of informal care to be 3 % of GDP for Sweden. However, they consider providing long-term care at a given point in time rather than those whose labour supply reduced after a deterioration in parental health and an increase in caregiving. As calculations on total costs of informal care are based on various assumptions, we decide to abstain from calculating total costs of informal care and, instead, focus on the costs resulting from labour supply reductions only, yielding more precise but lower GDP effect estimates.

This paper offers valuable insights for policymakers who aim to prepare their countries for increasing care needs in the future due to an ageing population while facing a shrinking labour force due to low birth rates and increases in life expectancy and increasing financial constraints. First, countries can support informal carers to better reconcile caregiving and work, preventing reductions in labour supply. Several countries have introduced paid and unpaid leave policies, where informal carers can take a short leave and return to their previous position afterwards (Rocard & Llena-Nozal, 2022). Evidence on the effect of these policies is limited, but where available, their impact prevented reductions in labour supply among informal carers (Coile, Rossin-Slater, & Su, 2022; Kim, 2020; Anand, Dague, & Wagner, 2022).

Second, policymakers can expand formal long term-care structures. This is of particular relevance for countries aiming to increase female labour market participation to compensate for a decline in the number of people in working age (Carrino, Nafilyan, & Avendano, 2022; He & McHenry, 2015). An increase in the availability of formal care can generally cushion the effect of a parental health deterioration on the labour market participation of daughters (Kolodziej, Reichert, & Schmitz, 2018). For example, (Hollingsworth et al., 2021) demonstrated that the introduction of free universal long-term care in Scotland through the 2002 Scottish Community Care and Health Act led to increases in labour market participation and the number of hours worked. Similarly, the lack of a noticeable effect of caregiving on labour supply in Norway was linked to its generous formal long-term care supply (Gautun & Bratt, 2024). In addition, expansions in long-term care benefits and supply have been found to reduce health expenditures, which was beyond the scope of this study (Costa-i-Font, Jimenez-Martin, & Vilaplana, 2018; Serrano-Alarcón, Hernández-Pizarro, López-Casasnovas, & Nicodemo, 2022).

Third, policymakers can also consider policies beyond the long-term care system alone. Investing in policies that support healthy ageing, such as physical activity and better diet, can directly reduce the degree and severity to which people become care-dependent in old age (Santos & Cylus, 2024; Bloom, et al., 2015). Investments in these measures are likely to increase in relevance in the near future. Population ageing will increase the need for long-term care, and at the same time reduce the share of population in working age. Additionally, a decline in the birth rate and low rates of siblings among caregivers (see Table 1) could place an increasing burden on caregivers as caregiving tasks can increasingly be shared among fewer children. As a result, the rate of caregivers that provide intensive forms of care could increase in the future, which increases the probability of reductions in labour supply compared to weekly caregivers that share their caregiving duties with siblings or formal carers from an official provider, or the undocumented, ‘grey’ market.

Our paper has several limitations that should be considered when interpreting our findings. First, we argue that the availability of formal care structures affects the degree to which women become informal carers and group countries based on their geographic characteristics. However, social norms, such as expectations on daughters to care for their parents, also shape their behaviour and can lead to different responses across countries despite similar generosities in long-term care benefits (Bakx, de Meijer, Schut, & van Doorslaer, 2014). Second, our study is limited to Continental European countries. The transferability to other country contexts should be approached with caution. However, studies from England and non-European countries, such as the United States, confirm our results (Carmichael & Charles, 2003; Maestas et al., 2024; Van Houtven et al., 2013). This increases our confidence that our findings hold in other country contexts as well. Third, we could underestimate the costs of informal caregiving, as we only estimate the effect for women aged 50–60. Despite being the most likely to provide informal care, they do not cover entire universe of informal carers. Further, we only look at people that dropped out of the labour force altogether (*extensive margin*) and could not take reductions in labour supply and productivity into account (*intensive margin*), although other scholars highlight these effects as well. Intensive caregiving affects the quality of life and well-being of carers (van den Berg, Brouwer, & Koopmanschap, 2004). It negatively affects mental health, leading to increased mental health costs among informal carers (Bom & Stöckel, 2021). Unfortunately, it is beyond the scope of this paper to quantify costs resulting from that, but we invite researchers to study such costs in an extension to this paper. Finally, we use a single estimate of the elasticity between employment and GDP for all the countries analysed. While these estimates are based on EU-zone countries, which constitute the majority of our sample, it is important to note that the elasticity represents the entire economy and may differ from that of our specific sample. However, the average wages in our sample are only moderately higher (approximately 22 %) than those in the broader economy, suggesting that the use of a single elasticity provides a reasonably good approximation for our analysis. Despite these limitations, we are confident that our results are informative to policymakers and researchers alike.

## Conclusion

Our results indicate a reduction in labour supply among women when they assume caregiving responsibilities. Responses are more pronounced in Western, Southern, and Central/Eastern European countries than Northern OECD countries, and there are considerable costs to societies attached to informal caregiving. Our results confirm existing research in showing a pronounced reduction in labour supply among women when they become caregivers. This leads to welfare losses to economies. Countries will have to decide whether they want to rely on informal carers to meet increasing demands from an ageing demographic or support the labour market participation of women to offset reductions in the share of people at working age.

## Figures and Tables

**Table 1:** Descriptive statistics of our sample.

	North	West	South	Centre/East

Workers (LP)	88 %	78 %	54 %	83 %
Daily caregivers (IC)	3 %	7 %	13 %	9 %
Weekly caregivers (IC)	18 %	21 %	22 %	21 %
Age	56.0	55.9	55.6	56.0
Married/partnership	73 %	70 %	82 %	71 %
Upper secondary or higher education	82 %	81 %	48 %	86 %
Poor health	1 %	2 %	3 %	3 %
Poor parental health (PH)	51 %	48 %	14 %	60 %
Number of children	2.5	2.2	1.9	2.3
Number of brothers	1.1	1.1	1.1	0.8
Number of sisters	1.1	1.1	1.0	0.8
Co-resident	0 %	2 %	9 %	8 %
Less than 5 kms	29 %	33 %	48 %	38 %
Nonwage income (median)	28.7	24.0	12.4	5.3
Number of observations	1,228	3,178	1,362	1,500
Number of unique observations	944	2,264	1,103	1,200

Source: Authors’ analysis. IC = Informal carers.

**Table 2:** Labour market supply by parental health and caregiving intensity.

	North	West	South	Centre/East

Both parents healthy	89 %	79 %	55 %	85 %
At least one parent in poor health	81 %	69 %	47 %	71 %
At least one parent in poor health & daily caregiver	58 %	66 %	54 %	66 %

Source: Authors’ analysis.

**Table 3:** Replication of pooled seemingly unrelated probit regression of Crespo and Mira (2014).

	Employment effect				Care effect				LATE			
	North	West	South	Centre/East	North	West	South	Centre/East	North	West	South	Centre/East

Model 1^(1)^	−0.063	−0.100	−0.072	−0.133	0.055	0.082	0.228	0.147	−1.144	−1.229	−0.316	−0.905
	(0.032)	(0.025)	(0.040)	(0.029)	(0.021)	(0.017)	(0.035)	(0.026)	(−3.957, −0.068)	(−2.341, −0.587)	(−0.745, 0.053)	(−1.536, −0.523)
Model 2^(2)^	−0.053	−0.086	−0.055	−0.122	0.051	0.075	0.216	0.135	−1.041	−1.137	−0.255	−0.898
	(0.030)	(0.023)	(0.038)	(0.029)	(0.021)	(0.018)	(0.035)	(0.025)	(−4.205, 0.170)	(−2.342, −0.487)	(−0.678, 0.117)	(−1.623, −0.474)
Model 3^(3)^	−0.034	−0.048	−0.063	−0.073	0.052	0.073	0.216	0.133	−0.665	−0.658	−0.292	−0.549
	(0.025)	(0.017)	(0.027)	(0.024)	(0.021)	(0.017)	(0.035)	(0.025)	(−2.629, 0.369)	(−1.397, −0.177)	(−0.626, −0.055)	(−1.099, −0.196)

Source: Authors’ analysis. Bootstrapped standard errors are in parentheses (employment and care effects). Brackets for LATE parameters represent a bootstrapped 95% confidence interval.

(1) With time fixed effects

(2) with age dummies, higher education dummy, number of sisters

(3) with lagged labour force participation.

**Table 4:** Pooled seemingly unrelated probit regression using OECD classification.

	Employment effect			Care effect			LATE		
	Cluster 1	Cluster 2	Cluster 3	Cluster 1	Cluster 2	Cluster 3	Cluster 1	Cluster 2	Cluster 3

Model 1^(1)^	−0.091	−0.096	−0.131	0.059	0.181	0.126	−1.545	−0.530	−1.043
	(0.023)	(0.023)	(0.050)	(0.016)	(0.021)	(0.039)	(−3.902, −0.730)	(−0.846, −0.258)	(−2.896, −0.246)
Model 2^(2)^	−0.080	−0.088	−0.123	0.056	0.175	0.113	−1.424	−0.501	−1.086
	(0.022)	(0.023)	(0.051)	(0.016)	(0.021)	(0.037)	(−3.478, −0.594)	(−0.830, −0.244)	(−3.170, −0.207)
Model 3^(3)^	−0.036	−0.071	−0.042	0.054	0.174	0.109	−0.664	−0.408	−0.385
	(0.016)	(0.019)	(0.034)	(0.016)	(0.022)	(0.037)	(−1.738, −0.083)	(−0.672, −0.186)	(−1.793, 0.212)

Source: Authors’ analysis. Bootstrapped standard errors are in parentheses (employment and care effects). Brackets for LATE parameters represent a bootstrapped 95% confidence interval.

(1) With time fixed effects

(2) with age dummies, higher education dummy, number of sisters

(3) with lagged labour force participation.

**Table 5:** Panel fixed-effect estimation based on typology by Crespo and Mira (2014).

	Employment effect				Care effect				LATE			
	North	West	South	Centre/East	North	West	South	Centre/East	North	West	South	Centre/East

Daily caregiving	−0.001 (0.055)	0.020 (0.031)	−0.103 (0.051)	−0.025 (0.032)	0.016 (0.048)	0.099 (0.029)	0.234 (0.074)	0.075 (0.041)	−0.040 (−14.163,17.149)	0.203 (−0.464,0.990)	−0.442 (−1.311,−0.008)	−0.338 (−3.714,1.253)
Weeklycaregi vi ng					−0.014 (0.070)	0.119 (0.039)	0.223 (0.079)	0.114 (0.063)	0.044 (−11.883,11.593)	0.169 (−0.417,0.924)	−0.463 (−1.783,0.000)	−0.222 (−1.886,1.145)

Source: Authors’ analysis. Bootstrapped standard errors are in parentheses (employment and care effects). Brackets for LATE parameters represent a bootstrapped 95% confidence interval. Controls include time fixed effects and daughter’s age.

**Table 6:** Estimated cost of informal caregiving.

	North	West	South	Centre/East

Affected population (%)	13 %	15 %	12 %	12 %
Affected population (N)	686,009	3,765,184	1,966,055	1,673,997
Employment effect	−0.13 %	−0.23 %	−0.28 %	−0.28 %
	(−0.33 %, 0.05 %)	(−0.39 %, −0.07 %)	(−0.53 %, −0.05 %)	(−0.45 %, −0.10 %)
GDP effect	−0.21 %	−0.37 %	−0.45 %	−0.44 %
	(−0.54 %, 0.07 %)	(−0.63 %, −0.11 %)	(−0.85 %, −0.08 %)	(−0.71 %, −0.17 %)

Source: Authors’ analyses. Brackets for employment and GDP effects represent a bootstrapped 95% confidence interval.

## Footnotes

1 We do not use wave 3 as it focuses on people’s life histories and wave 7 due to the missing observation on parents’ health.

2 Countries in accession to the OECD.

3 Parental health is classified as poor if the child reports it as poor (or very poor in wave 1). Otherwise, it is classified as good.

4 A child is classified as a daily caregiver if they report providing help to their parents almost daily in the last twelve months. A child is classified as a weekly caregiver if they report providing help to their parents either almost daily or almost every week in the last twelve months.

5 GDP data has been downloaded from the website of the OECD and is collected based on in accordance with the System of National Accounts 2008, which excludes informal care from the GDP (European Commission, International Monetary Found, Organisation for Economic Co-operation and Development, United Nations, World Bank, 2009).

## Annex A. Methodology of long-term care classification

The OECD methodology uses a comprehensive clustering analysis to classify long-term care systems based on five dimensions, providing a more through and diverse assessment. This approach improves upon previous typologies, which used fewer indicators and lacked coverage in areas such as availability and informal care support (Ariaans et al., 2021; Kraus et al., 2010). Additionally, the use of set of algorithms ensures the stability and robustness of country classifications. Llena-Nozal et al. (2025) contains the full description of the methodology of long-term care classification.

### Data

We conduct the information and data collection process along the following five comparative dimensions: governance and organisation; funding; quality; availability; and access. The data used in this analysis comes from OECD indicators of long-term care, from OECD questionnaires on long-term care and from relevant literature and covers a total of 27 countries.

Incorporating a dimension focused on the governance of public long-term care provision helps to understand the organisational depth and cohesion of long-term care systems. Four variables are used to classify countries. The first variable assesses whether countries have legislation that unifies the provision of benefits and services under long-term care, considering that these may fall within the responsibilities of both health and social policy ministries. The second variable evaluates the level of decentralisation within long-term care systems. The third variable captures the extent of public versus non-public provision of long-term care, taking into account the percentage of providers that are public compared to those that are private for-profit or private non-profit. Finally, the fourth variable measures the degree of integration between the long-term care sector and the health care sector, particularly through the use of guidelines, care pathways, and multidisciplinary teams that connect the two systems.

The funding dimension seeks to capture the extent of public funding and cost-sharing across countries, using three key variables. The first variable assesses the proportion of long-term care costs covered by public funds for an older person with severe needs and a median income (see OECD (2024)) for detail description of typical cases). The second variable measures the out-of-pocket expenses for long-term care as a percentage of median income for a person with severe needs. Lastly, the third variable evaluates the impact of social protection on poverty reduction among older individuals with severe needs.

The quality dimension evaluates various aspects of long-term care quality using five key variables. The first variable examines whether staff-to-resident ratios are mandated in the countries. The second assesses the quality of staffing by determining if minimum education requirements exist for long-term care workers. The third and fourth variables focus on quality assurance and regulation: the third measures whether mandatory accreditation is required for institutions, home care services, or both, while the fourth checks for the presence of a quality assurance framework within the long-term care system. Finally, the fifth variable assesses care outcomes.

The availability dimension focuses on the supply of formal care and the support for family carers. It first examines the supply of care through a proxy, which includes the number of beds and the total number of long-term care workers (both in home and institutional settings) relative to the population aged 65 and above. Secondly, this dimension assesses reliance on and support for family carers by creating a familialism variable, which considers the existence of carer leave and cash benefits. This variable is coded from 0 to 4, depending on whether all four types of support (cash benefits for carers, cash benefits for care recipients that can be used for carers, paid leave, and unpaid leave) are available. Additionally, another variable measures the reliance on informal carers, specifically the percentage of care provided by informal carers.

The access dimension examines how well current systems meet needs, the degree of targeting, and whether countries rely on in-kind services or cash benefits. The first and second variables assess the level of targeted access based on needs and income, comparing out-of-pocket expenditures for more disadvantaged groups. The third variable calculates a coverage rate by comparing the share of individuals aged 65 + with long-term care needs to the share of recipients among this age group, using ADLs, IADLs, and, where possible, the OECD measure of needs based on typical cases. The fourth variable evaluates the extent to which a country provides services directly through in-kind services, relies solely on cash benefits, or offers a choice between in-kind services and cash benefits, assigning the highest value to the most comprehensive option.

### Methodology

In the first step, we apply Principal Component Analysis (PCA) to reduce the dimensionality of the data, addressing the high number of variables while maximizing interpretability and preserving as much variation as possible. In the second step, we implement multiple clustering algorithms to classify the countries. Given that each clustering method has its own strengths and weaknesses, and no single method consistently outperforms the others, we synthesize the results from the different algorithms to derive the final classification. We assign each country to the cluster in which it most frequently appears across the various methods.

To classify countries, we employ four clustering algorithms, which are categorised into two main types: distance-based clustering and probabilistic clustering. Distance-based clustering involves grouping data points into clusters based on their similarity, determined using a distance metric. The core principle is to cluster together data points that are close to each other in the feature space, thereby reflecting their proximity. For this purpose, we employ three algorithms that fall within the distance-based category. Each algorithm employs a unique method for measuring similarity and grouping data points.

The first algorithm we employ is k-means clustering (Lloyd, 1982), one of the most widely used clustering methods. It works by assigning data points to clusters in such a way that the distance between each data point and the centroid of its assigned cluster is minimized. Initially, the centroids of a specified number of clusters are randomly selected. While the algorithm’s simplicity is a major advantage, it is sensitive to the initial random selection of centroids, which can lead to varying results. Additionally, determining the optimal number of clusters is challenging, as there is no universal theoretical solution for this problem.

The second algorithm we use is hierarchical clustering with the Ward method of linkage (Ward, 1963), which organizes similar data points into clusters that form a hierarchical structure. This structure reflects the order in which clusters are merged or divided, providing a comprehensive view of the clustering process. Unlike k-means clustering, hierarchical clustering does not require the specification of the number of clusters in advance, and it is not sensitive to the initial selection of cluster centroids. However, it can be sensitive to outliers, and the results may be influenced by the choice of distance metric and linkage method used.

The last distance-based algorithm we employ is the Self-Organizing Map (SOM), a type of artificial neural network developed to reduce the dimensionality of data while preserving the topological relationships between data points (Kohonen, 1982). This algorithm is particularly robust against noise and outliers within the data, making it a reliable choice in complex datasets. However, SOM can be sensitive to the initial configuration of neurons, and its performance is highly dependent on the selection of parameters such as grid size, learning rate, and neighbourhood size, which can influence the final outcome.

The second group of algorithms we use are probabilistic clustering methods, which assign data points to clusters based on probabilistic models or probability distributions. Unlike distance-based methods, these algorithms calculate the likelihood that each data point belongs to a particular cluster, allowing for more flexible and nuanced cluster assignments. This approach is particularly advantageous when data points may belong to multiple clusters or when there is uncertainty in the cluster assignments, as it accommodates overlapping clusters and provides a probabilistic understanding of the data’s structure.

Among the probabilistic clustering methods, we employ the Gaussian Mixture Model (GMM). This method assumes that the data points are generated from a mixture of several Gaussian distributions, with each distribution representing a cluster. GMM utilizes the Expectation-Maximization technique to estimate the parameters, such as the mean and covariance, for each of these distributions. This allows for the calculation of the probability that each data point belongs to a given cluster, making GMM more flexible compared to k-means clustering. However, GMM lacks a universal theoretical framework for determining the optimal number of clusters and is sensitive to initial parameter estimates, which can influence the final clustering outcome.

Finally, before constructing clusters, we need to determine the number of clusters, as most clustering algorithms (with the exception of hierarchical clustering) require this information beforehand. As previously mentioned, there is no universal theoretical solution for determining the optimal number of clusters. However, the literature provides various heuristics, such as the elbow method commonly used for k-means algorithms, to guide this selection. By considering these heuristics alongside the stability of cluster assignments across different methods, we have decided to set the number of clusters to three. This choice balances both the interpretability and robustness of the clustering results.

### Comparison of typologies by OECD and by Crespo and Mira (2014)

Table 7 compares the classification of countries based on the OECD typology with the typology proposed by Crespo and Mira (2014). Cluster 1 includes countries categorised as part of the North and West in Crespo and Mira’s typology. Cluster 2 comprises a mixture of countries from the West, South, and Centre/East regions. Within Cluster 2, countries from the South and Centre/East regions generally exhibit more developed long-term care systems compared to countries from these regions classified in Cluster 3. Cluster 3 primarily consists of Centre/East countries and Greece which is part of the South. The strong correlation between geographic clustering and the OECD typology suggests that the development of long-term care systems is closely tied to the social norms and cultural characteristics prevalent in each geographic region.

**Table 7:** Clustering of countries by OECD, and Crespo and Mira’s (2014) typology.

	North	West	South	Centre/East

Cluster 1	Denmark, Finland, Netherlands, Sweden	Austria, Belgium, Germany, Luxembourg		
Cluster 2		France	Italy, Spain	Czechia, Estonia, Hungary, Lithuania, Slovakia
Cluster 3			Greece	Bulgaria, Croatia, Latvia, Poland, Romania, Slovenia
Not clustered		Switzerland		

Source: Authors’ analysis. Switzerland is not clustered in the OECD typology due to the data limitation.

## Annex B. Additional descriptive statistics and results

**Table 8:** Intensive caregiving by gender.

	North	West	South	Centre/East

Men	1.1 %	3.0 %	4.6 %	7.4 %
Women	2.6 %	6.6 %	13.4 %	9.3 %

Source: Authors’ analysis.

**Table 9:** Descriptive statistics by parental health and caregiving intensity.

	Both parents healthy	At least one parent in bad health	At least one parent in bad health & daily caregiver

Age	55.80	56.25	56.51
Married/partnership	73 %	73 %	75 %
Upper secondary or higher education	76 %	75 %	72 %
Poor health	1.6 %	4.2 %	3.1 %
Number of children	2.22	2.22	1.98
Number of brothers	1.03	1.04	0.77
Number of sisters	1.03	0.99	0.77
Living less than 5 kms away	4 %	7 %	25 %

Source: Authors’ analysis.

**Table 10:** OLS estimation based on typology by Crespo and Mira (2014).

	Employment effect				Care effect				LATE			
	North	West	South	Centre/East	North	West	South	Centre/East	North	West	South	Centre/East

Model 2^1^	−0.058	−0.088	−0.052	−0.124	0.055	0.077	0.215	0.143	−1.044	−1.141	−0.242	−0.870
	(0.030)	(0.023)	(0.036)	(0.030)	(0.022)	(0.017)	(0.035)	(0.025)	(−4.413, 0.086)	(−2.332, −0.541)	(−0.609, 0.113)	(−1.557, −0.458)
Model 3^2^	−0.035	−0.050	−0.058	−0.080	0.055	0.076	0.215	0.141	−0.637	−0.657	−0.268	−0.568
	(0.027)	(0.018)	(0.026)	(0.024)	(0.020)	(0.017)	(0.034)	(0.026)	(−2.350, 0.365)	(−1.442, −0.208)	(−0.569, −0.035)	(−1.081, −0.239)

Source: Authors’ analysis. Bootstrapped standard errors are in parentheses (employment and care effects). Brackets for LATE parameters represent a bootstrapped 95 % confidence interval.

(1) With age dummies, higher education dummy, number of sisters

(2) with lagged labour force participation.

**Table 11:** Panel estimation based on OECD typology.

	Employment effect			Care effect			LATE		
	Cluster 1	Cluster 2	Cluster 3	Cluster 1	Cluster 2	Cluster 3	Cluster 1	Cluster 2	Cluster 3

Dailycaregiving	0.027 (0.035)	−0.048 (0.030)	−0.049 (0.060)	0.055 (0.030)	0.150 (0.036)	0.113 (0.106)	0.490 (−1.711,4.029)	−0.320 (−0.887,0.094)	−0.437 (−5.803,3.473)
Weeklycaregiving				0.092 (0.043)	0.141 (0.043)	0.279 (0.137)	0.293 (−1.409,2.028)	−0.340 (−1.115,0.125)	−0.177 (−1.368,0.350)

Source: Authors’ analysis. Bootstrapped standard errors are in parentheses (employment and care effects). Brackets for LATE parameters represent a bootstrapped 95 % confidence interval. Controls include time fixed effects and daughter’s age.
